# Mitigating the affective and cognitive consequences of social exclusion: an integrative data analysis of seven social disconnection interventions

**DOI:** 10.1186/s12889-024-18365-5

**Published:** 2024-05-07

**Authors:** Randy T. Lee, Gizem Surenkok, Vivian Zayas

**Affiliations:** Department of Psychology, Cornell University, Ithaca, NY USA

**Keywords:** Social exclusion, Loneliness, Social isolation, Interventions, Buffering, Recovery, Friendships

## Abstract

**Background:**

Being socially excluded has detrimental effects, with prolonged exclusion linked to loneliness and social isolation. Social disconnection interventions that do not require direct support actions (e.g., “how can I help?”) offer promise in mitigating the affective and cognitive consequences of social exclusion. We examine how various social disconnection interventions involving friends and unknown peers might mitigate social exclusion by buffering (intervening *before*) and by promoting recovery (intervening *after*).

**Methods:**

We present an integrative data analysis (IDA) of five studies (*N* = 664) that systematically exposed participants to exclusion (vs. inclusion) social dynamics. Using a well-validated paradigm, participants had a virtual interaction with two other people. Unbeknownst to participants, the other people’s behavior was programmed to either behave inclusively toward the participant or for one to behave exclusively. Critically, our social disconnection interventions experimentally manipulated whether a friend was present (vs. an unknown peer vs. being alone), the nature of interpersonal engagement (having a face-to-face conversation vs. a reminder of an upcoming interaction vs. mere presence), and the timing of the intervention in relation to the social dynamic (before vs. during vs. after). We then assessed participants’ in-the-moment affective and cognitive responses, which included mood, feelings of belonging, sense of control, and social comfort.

**Results:**

Experiencing exclusion (vs. inclusion) led to negative affective and cognitive consequences. However, engaging in a face-to-face conversation with a friend *before* the exclusion lessened its impact (*p* < .001). Moreover, a face-to-face conversation with a friend *after* exclusion, and even a reminder of an upcoming interaction with a friend, sped-up recovery (*p*s < .001). There was less conclusive evidence that a face-to-face conversation with an unknown peer, or that the mere presence of a friend or unknown peer, conferred protective benefits.

**Conclusions:**

The findings provide support for the effectiveness of social disconnection interventions that involve actual (i.e., face-to-face) or symbolic (i.e., reminders) interactions with friends. These interventions target momentary vulnerabilities that arise from social exclusion by addressing negative affect and cognitions before or after they emerge. As such, they offer a promising approach to primary prevention prior to the onset of loneliness and social isolation.

**Supplementary Information:**

The online version contains supplementary material available at 10.1186/s12889-024-18365-5.

The importance of social connection has garnered global attention from policy makers, health professionals, and researchers [1]. Citing the well-documented consequences of loneliness, including that it is as harmful as obesity, increases the risk of stroke and type 2 diabetes, and is as bad as smoking 15 cigarettes per day, governments worldwide, including Australia, Japan, South Korea, the United Kingdom, and the United States [2–5], have taken measures to combat the global loneliness epidemic. These measures include implementing national strategies to combat loneliness to paying people to re-enter society. Recognizing that young people are more likely to report experiencing loneliness than older people [6–10], one area of focus in the global effort to combat loneliness and social isolation has been on supporting young people—a group who is “at a high risk of feelings of loneliness but are least likely to take action” [11].

The Romantic-era poet, critic, and philosopher Samuel Taylor Coleridge wrote that “friendship is a sheltering tree” [12]. Just as a tree provides shade and shelter from the elements, Coleridge’s metaphor highlights how friendships can protect and support individuals amidst life’s challenges and uncertainties by serving as a source of protection, comfort, and security. We contend that this sentiment provides an astute insight into the power social support can offer in the face of threats to social connection.

One pervasive threat to social connection arises from social exclusion, a common occurrence in day-to-day life [13], as it is not possible for everyone to be included in all events at all times. Social exclusion, loneliness, and social isolation are distinct, yet interrelated constructs [14–23]. Social exclusion refers to a broad category that encompasses any negative interpersonal encounter where one is left out or ostracized; loneliness refers to the subjective feeling of being alone or the perception of deficiencies in the quality or quantity of social relationships; and social isolation refers to an objective state characterized by limited interaction and detachment from social groups and activities. All three arise from unmet social needs (whether subjective or objective) and share common risk factors and outcomes [24–28]. Most importantly, they mutually reinforce each other, resulting in a vicious cycle of social disconnection.

Previous work has suggested two promising routes for social disconnection interventions aimed at alleviating vulnerability for loneliness. First, interventions should focus on increasing opportunities for social contact [29]. Diverse approaches, such as promoting group activities, have been used to enhance the features of people’s social environments so that they are more likely to have social interactions. However, the precise features of social disconnection interventions that are most effective remain unexplored. In particular, it is unclear whether interventions need to involve deep and meaningful interactions or if even superficial and routine exchanges, or if the mere social presence of peers, can be beneficial.

Second, social disconnection interventions should focus on cognitions that lead to social avoidance and withdrawal [30]. Accordingly, interventions have aimed at helping individuals change potentially problematic ways of thinking. Still, there has been less attention directed towards how social interventions may serve as a primary prevention strategy by reducing the activation of cognitions that would lead to further social withdrawal and isolation.

## Primary prevention in combatting risk for loneliness and social isolation

The present work examines the extent to which seven social disconnection interventions may serve to decrease the risk of loneliness and social isolation. Building on the broad literature on social exclusion, loneliness, and social isolation, we propose that transient states of social disconnection that arise from everyday situations can pose *momentary vulnerabilities* for developing more persistent feelings of loneliness. Social exclusion (actual or imagined) can lead to lowered affect, self-esteem, sense of control, and meaningful existence. Such responses to social exclusion could lead to attempts for reconnection and prosocial tendencies. But, when not handled properly, they can lead to withdrawal and eventually more persistent feelings of social disconnection, resulting in chronic feelings of loneliness [14–23]. Thus, negative affect and cognitions that arise from instances of social exclusion are a crucial entry point for interventions. Social disconnection interventions that target *momentary vulnerabilities* present a promising primary prevention strategy [31, 32]. They promptly address negative affect and cognitions before, or immediately after they arise, prior to the onset of loneliness and social isolation.

Our social disconnection interventions focus on the individuals involved in the intervention (friend, unknown peer, or alone), the nature of interpersonal engagement (an actual face-to-face conversation, anticipated interaction, friend in game, or mere presence), and the timing of the social disconnection intervention (before, during, or after an exclusion episode).

### Do the individuals involved in the social disconnection intervention make a difference?

Social ties serve essential self- and social-regulatory functions at the levels of affect, cognition, behavior, and physiology [33–47], making them fundamental to well-being [48–50]. As a highly social species, humans have inherited cognitive architecture that makes affiliation, or even the promise of it, rewarding [51].

One particularly important type of social tie is friendships. Not only do friends provide pleasure, companionship, and intimacy, they stimulate social and intellectual development [52–54]. Correlational work finds that the number and quality of friendships are associated with greater self-reported well-being and self-esteem [55–58]. The number of friends one has predicts health, happiness, and academic, financial, and professional success [52, 59–61]. We propose that friendships may be particularly instrumental in protecting individuals from social disconnection through their affective and cognitive regulatory functions. Although affective and cognitive regulatory functions are typically associated with an individual’s closest, deepest attachment bonds—such as those with a primary caregiver in early life and a romantic partner in adulthood—friends serve similar functions, though with muted intensity, frequency, specificity, and persistency [53, 54, 62–68]. Specifically, friends can restore feelings of comfort and security following threats, and can confer confidence to explore new environments. Supporting these ideas, developmental research shows that by middle childhood, children increasingly turn to their friends for emotional support, by late adolescence, peers are often preferred over parents as sources of comfort, and in adulthood, friends remain a source of comfort and security [53, 63, 64, 69, 70].

People are able to identify who they turn to for specific affective regulatory needs (e.g., who do you turn to to cheer you up when you are feeling sad?) and having a diverse social network not only facilitates this process, but is associated with well-being [71, 72]. While people are more likely to seek support from those they perceive as being closer [73], some work suggests that even unknown others (i.e., strangers) may also provide affective and cognitive benefits. Indeed, social interactions with peripheral members of social networks and strangers have the power to increase feelings of belongingness [74–76]. Moreover, interactions with unknown others can modulate physical pain, reducing pain intensity and feelings of unpleasantness while fostering feelings of social support (see [77] for a review). This literature suggests the possibility that strangers may also confer protection against the negative consequences of social disconnection. Thus, in our research, we examine social disconnection interventions that involve friends and unknown peers. Additionally, we investigate how relationship quality with the friend may be related to the efficacy of social disconnection interventions specifically involving friends.

### What about the nature of interpersonal engagement of the social disconnection intervention?

Previous research has found that friendly interactions can mitigate the affective and cognitive consequences of common life stressors [78]. Even the mere thought of another person can help. Thinking about an attachment figure (such as a close friend or family member) can help to regulate negative emotions and cognitions triggered by stress [79–81]. However, the mere presence of another may not always be enough in the face of social disconnection. Research using experience sampling finds that feelings of social disconnection can actually be *amplified* when others are simply present [82]. In our research, we examine the nature of interpersonal engagement (e.g., engaging in a conversation vs. being reminded of an upcoming interaction vs. simply being in the presence of another) to provide clarity to the seemingly divergent findings in the existing literature.

### Does the timing of the social disconnection intervention matter?

A social disconnection intervention *before* social exclusion may *buffer* against the affective sting and cognitive reactions of exclusion. Conversely, a social disconnection intervention *following* social exclusion may promote *recovery* from the negative consequences of exclusion. With regard to buffering, perhaps surprisingly, there is mixed experimental evidence that friendships *buffer* against adverse social experiences [81, 83–85]. Moreover, of the studies that do find some evidence, buffering effects are usually weak [83]. In contrast, studies using *recovery* interventions generally find stronger and more robust effects of social presence on reducing the affective sting and cognitive reactions of social exclusion [86, 87]. Indeed, even mundane interactions with an unknown peer can promote recovery following exclusion [88]. In our investigation, we systematically examine whether the timing of social disconnection interventions matters.

### Overview of the present work

The current work aims to understand how friends, and even the mere presence of an unknown peer, may mitigate the negative consequences of being excluded or ignored in a social setting, a common form of social exclusion that may be a precursor to chronic loneliness and social isolation [14–23]. Across five studies featuring seven distinct social disconnection interventions, we systematically examine how the affective sting and cognitive reactions of social exclusion are dampened by various forms of social presence. All studies were part of the same project and participants were restricted from participating in more than one study. We focused on social disconnection interventions that are likely to occur naturally in day-to-day life (e.g., a conversation that occurs prior to or following a negative event). We instantiated the experience of social exclusion using a modified version of Cyberball, a well-validated, virtual ball-tossing game used to experimentally manipulate exclusion that reliably induces negative affect and threatens four fundamental needs (i.e., self-esteem, feelings of belonging, meaningful existence, and control) (see Hartgerink et al., (2015) for a meta-analysis) [89].

Critically, across the five studies, we varied three features of the social context during which the exclusion took place: (1) whom participants interacted with (alone vs. an unknown peer vs. a friend), (2) the nature of interpersonal engagement (engaging in a face-to-face, in-person conversation vs. being reminded of an upcoming interaction), and (3) the timing of the social presence relative to the experience of social exclusion (before vs. during vs. after). Our goal was to investigate how specific features of social presence serve to either protect individuals from the negative effects of exclusion (i.e., buffering), retroactively help in the restoration of affect and fundamental needs following the effects of exclusion (i.e., recovery), or both. We utilized integrative data analysis (IDA) because the designs of all five of the studies were highly similar and examined the same dependent variable.^1^

IDA is the statistical analysis of pooled data and is ideal when the original individual data from multiple studies are available. IDA approaches differ from and offer advantages over meta-analytic techniques, which also have the goal of building a cumulative knowledge base. Specifically, IDA approaches pool the original raw data, allowing researchers to examine what works, for whom, and in which contexts. This is in contrast to meta-analysis, which provide a synthesis of summary statistics drawn from multiple studies (see Curran & Hussong (2009) and Hussong, Curran, & Bauer (2013) for discussions) [90, 91]. IDA has been applied in medical research to assess treatment efficacy such as the comparison between medications and cognitive-behavioral therapy for severe depression [92]. Similarly, in the field of personality research, IDA has been utilized to explore the links between personality and life outcomes [93].

Our approach draws parallels with research in medicine. For example, in the study of skin wound healing, investigators create a small abrasion and implement interventions to observe how the body responds [94, 95]. Similarly, we use and adopt an experimental social psychological lens, manipulating social disconnection interventions before or after exposing participants to a form of social exclusion that effectively induces feelings of social disconnection.

To our knowledge, no study has systematically investigated the extent to which various forms and timing of social presence of friends influences the affective and cognitive reactions triggered by social exclusion.

## Method

Participants were run in one of five studies, with each study utilizing different social disconnection interventions to examine how social presence shapes the affective experience of social exclusion. Table 1 presents an overview of all five studies, specifying the conditions and social disconnection interventions employed in each.

**Table 1:** Overview of all five studies, specifying the conditions and social disconnection interventions employed in each

Social Disconnection Interventions		Study				
		Study 1	Study 2	Study 3	Study 4	Study 5
**Comparison**	Alone (Inclusion)	✓				
	Alone (Exclusion)	✓				
**Buffering**	Mere presence of an unknown peer (Exclusion)		✓			
	Mere presence of a friend (Inclusion)			✓		
	Mere presence of a friend (Exclusion)		✓	✓		✓
	Conversation with an unknown peer before (Exclusion)		✓			
	Conversation with a friend before (Exclusion)					✓
	Friend is in the same game (Inclusion)			✓		
	Friend is in the same game (Exclusion)			✓		
**Recovery**	Reminder of an upcoming interaction with a friend after (Inclusion)				✓	
	Reminder of an upcoming interaction with a friend after (Exclusion)				✓	
	Conversation with a friend after (Exclusion)					✓

Note: Inclusion and exclusion refer to whether the participant experienced social inclusion or exclusion in the Cyberball game. See Table 3 for detailed descriptions of each condition

### Participants and design

Six hundred sixty-four participants (*M*_age_ = 20.29, *SD*_age_ = 2.29) were recruited to participate from Cornell University. Participants self-identified as 64.5% Female; 55.4% White, 30.8% Asian, 7.7% Black, 4.2% Latino, 4.2% Other.^2^ See Table 2 for a breakdown of demographics by Study. Participants who completed the study with a friend reported a mean friendship length of 19.76 months (*SD* = 19.51; *Range* = 1–150) with that specific friend. Participants were compensated for their involvement, and could choose to receive either course credit or a payment of $5.

**Table 2:** Breakdown of Demographics by Study

Study	*N*	*M*_age_ (*SD*_age_)	Gender	Race and Ethnicity
1	85	21.18 (4.52)	61.2% Female, 28.2% Male	50.0% White, 33.8% Asian, 16.2% Black, 6.8% Latino, 4.1% Other
2	177	19.46 (1.18)	69.5% Female, 29.4% Male	66.7% White, 23.2% Asian, 13.0% Black, 8.7% Latino, 1.4% Other
3	206	19.81 (1.50)	60.2% Female, 39.8% Male	53.7% White, 36.9% Asian, 5.9% Black, 1.0% Latino, 2.5% Other
4	96	20.09 (1.75)	64.6% Female, 35.4% Male	65.6% White, 21.9% Asian, 4.2% Black, 3.1% Latino, 5.2% Other
5	100	19.98 (1.86)	67.0% Female, 33.0% Male	49.0% White, 31.0% Asian, 4.0% Black, 7.0% Latino, 9.0% Other
Combined	664	20.29 (2.29)	64.5% Female, 33.9% Male	55.4% White, 30.8% Asian, 7.7% Black, 4.2% Latino, 4.2% Other

Study 1 is missing demographics for 9 participants due to a coding issue and participants not answering. Study 2 is missing gender information for 2 participants due to participants not answering, and is missing race and ethnicity information for 106 participants due to a coding issue and participants not answering. Study 3 is missing age and race and ethnicity information for 3 participants due to participants not answering. The total percentage exceeds 100% because participants were able to select multiple identities

Below, we provide a broad overview of the social exclusion paradigm, the recruitment strategy, and then the key dependent variable that we used to examine affective and cognitive consequences. Then, we provide a description of each of the five studies, detailing the exact nature of each intervention and unique and shared experimental features (e.g., social presence of a close friend), in Table 3.

**Table 3:** Description of the social disconnection interventions and comparison conditions

Social Presence	Study	Description
**Comparison**		
Alone	1	No other participants were in the experimental room during the study.
**Buffering Intervention**		
Mere presence of an unknown peer	2	During the game, an unknown peer was in another private cubicle in the same experimental room.
Mere presence of a friend	2, 3, 5	Participants did not play in the same game as their friend (i.e., none of the players had their friend’s name). During the game, friend was in another private cubicle in the same experimental room.
Conversation with an unknown peer before	2	Based on the pretest, participants were told that they were “very compatible” with their partner (a confederate), with whom they had a five-minute structured “getting to know you” interaction (e.g., “What is your name?”, “What year are you?”, “What are you majoring in?”) before the game. During the game, the previously unknown peer was in another private cubicle in the same experimental room.
Conversation with a friend before	5	Before playing game, participants had an interaction with their friend using “Fast Friends” questions. Participants did not play in the same game as their friend (i.e., none of the players had their friend’s name). During the game, friend was in another private cubicle in the same experimental room.
Friend is in the same game	3	Participants were explicitly told that their friend was playing the same game as them (i.e., the name of one of the players in the game was their friend’s name). During the game, friend was in another private cubicle in the same experimental room.
**Recovery Intervention**		
Reminder of an upcoming interaction with a friend after	4	After playing game, participant was reminded of an upcoming interaction with their friend. During the game, participants did not play in the same game as their friend (i.e., none of the players had their friend’s name). During the game, friend was in another private cubicle in the same experimental room.
Conversation with a friend after	5	During the game, participants did not play in the same game as their friend (i.e., none of the players had their friend’s name). During the game, friend was in another private cubicle in the same experimental room. After playing game, participants had an interaction with their friend using “Fast Friends” questions.

Table 3 is organized such that the social disconnection interventions are listed from low social presence to high social presence. For example, participants in the “Alone” conditions (our comparison conditions) played Cyberball with no other participant in the room. “Fast Friends” questions (e.g., “*What would constitute a perfect day for you?*”) are taken from Aron and colleagues [102]. Participants who completed the study with a friend were recruited to participate with a friend. Those who did not were recruited alone. All participants completed the study in the privacy of their own cubicle. See Supplemental Materials Document for more details about the methods for each study in our integrative data analysis

### Procedure and materials

#### Overview

Participants were recruited to either participate alone (Study 1, Study 2) or with a friend (Study 2, Study 3, Study 4, Study 5). All participants were told that they would “be playing an online game, in addition to completing a few, brief questionnaires.” For both recruitment methods, we utilized a multipronged approach, which included the use of listservs, classroom advertisements, and flyers. This comprehensive strategy aimed to maximize outreach and facilitate a varied participant pool. We aimed to recruit as many participants as possible based on time and funding constraints. The number of participants was influenced by the number of friend pairs who signed up.

Before coming to the lab, participants completed a brief pre-test survey that included demographics. All participants completed the study in the same room, either seated in a separate cubicle or partitioned table. In this manner, we ensured that participants could not see the computer screens of the other participants.

After participants were seated and asked to sign a consent form, they were randomly assigned to an experimental condition that manipulated social presence (see Table 1 and Table 3). To increase the believability of the cover story that participants would be playing the game with other people, the experimenter left the room for 2 minutes to “check on the other players.” Upon the experimenter’s return, participants were asked to put on headphones that had white noise playing “to block the sound of other participants also working on this experiment.” Participants were then told that they would be playing a ball tossing game (i.e., Cyberball) [96–98] and that “each player will have the opportunity to throw and toss the ball to each of the other players. When the ball is tossed to you, you may click on the name of the player you want to throw it to.” After playing Cyberball, participants completed a measure to assess affective and cognitive state (i.e., mood, sense of belonging, feelings of control, social comfort), which served as our primary outcome measure. At the end of the experiment, participants were probed for suspicion, debriefed on the purpose of the study, and thanked for their time.

#### Cyberball

We used a modified version of Cyberball [96–98] administered using Inquisit 3.0.5.0 [99] that instantiated the experience of inclusion or one-person exclusion [96–98]. In the inclusion condition, both players threw the ball 50% of the time to the participant and 50% of the time to the other player. In the one-person exclusion condition, one player never threw the ball to the participant while the other player threw the ball to the participant 50% of the time. This social exclusion dynamic has been shown to elicit negative affect and lower fundamental needs to the same extent as social exclusion instantiated by two players in the game [96–98].

#### Social disconnection intervention

In our social disconnection interventions, we varied (1) whom participants interacted with (alone vs. an unknown peer vs. a friend), (2) the nature of interpersonal engagement (a face-to-face conversation vs. a reminder of an upcoming interaction vs. mere presence),^3^ and (3) the timing of the social presence relative to the experience of social exclusion (before vs. during vs. after). Additionally, we examine the possibility that relationship quality (i.e., perceived closeness, perceived familiarity, perceived similarity, and feelings of positivity) is associated with affective and cognitive state for social disconnection interventions that involve friends.^4^

To examine the possibility that the simple presence of an unknown peer can affect people’s experience of exclusion, we recruited participants to take part in the study alone (i.e., with no other participants present at the same time). Participants in the *alone* conditions experienced exclusion or inclusion dynamics, and these conditions served as benchmarks from which to assess the effect of our social disconnection interventions. Indeed, in typical Cyberball studies, although participants are run in “private” cubicles, there are still other participants in the same room or lab space (e.g. [100, 101]). Thus, it is possible that the other participants may provide some benefits. Our design allowed us to assess whether the presence of any peer provides benefits as compared to when participants are completely alone. See Table 3 for a description of the social disconnection interventions and our comparison conditions.

#### Affective and cognitive state

Similar to previous work [103–105], we assessed affective and cognitive state with a 12-item questionnaire measured on a 7-point semantic differential bipolar scale. Each item captures a spectrum between two contrasting points, expressed through opposite adjectives or phrases. The questionnaire covers mood (“*sad/happy*,” “*friendly/unfriendly*,” “*angry/pleasant*”), belonging (“*disconnected/connected*,” “*I belong/I don’t belong*,” “*like an outsider/like an insider*”), control (“*powerless/powerful*,” “*I have control/I lack control*,” “*uninfluential/influential*”), and social comfort (“*uneasy/at ease*,” “*comfortable/uncomfortable*,” “*awkward/not awkward*”). Higher scores indicate greater levels of mood, sense of belonging, perceived control, and social comfort. Because Cronbach’s alpha was above .79 for each subscale and above .80 for the composite score, we computed a composite score to represent affective and cognitive state.

#### Relationship quality

Participants who participated in the study with a friend were asked to answer questions about the quality of their relationship with said friend.^5^ This questionnaire asked participants to consider their relationship with the friend and answer questions about familiarity (“*How familiar are you with this person?*”), significance (“*Is this person personally significant to you?*”), closeness (“*How close do you feel with this person?*”), and feelings of positivity (“*How* *strong* *are your POSITIVE feelings about this person?*”). The response scale was presented as a continuum without explicit numerical labels. ‘*Not at all’* represented the lower end of the scale, *‘Little’* represented the lower-midpoint, ‘*Somewhat’* represented the midpoint, ‘*Very’* represented the upper-midpoint, and ‘*Extremely’* represented the upper end of the scale. Cronbach’s alpha for these items was .89. Therefore, a composite score composed of familiarity, closeness, significance, and positivity was used to represent relationship quality.

#### Data Analytic Strategy

Given that the methodology across the five studies was highly similar, we present an integrative data analysis (IDA). Specifically, we capitalized on the large pooled data set to examine how different social disconnection interventions shape affective and cognitive experiences of social exclusion. To account for the nested structure inherent to pooled data, we utilized multilevel modeling (MLM). MLM is particularly suited for the analysis of nested data, where participants are grouped within studies. We ran two sets of MLM models where we treated each study as a random factor, and each participant was nested within each study. The first set of MLMs focused on assessing buffering interventions—i.e., evaluating each social disconnection intervention’s potential to proactively or concurrently mitigate the effects of social exclusion on affective and cognitive state. The second set of MLMs focused on assessing recovery interventions—i.e., evaluating each social disconnection intervention’s potential to assist in alleviating the negative affective impact following social exclusion. In all MLMs, our main dependent variable, *affective and cognitive state*, was assessed following the social dynamic. To assess the effect of specific social presence interventions, we computed contrast codes (e.g., exclusion alone vs. presence of friend) and entered the contrast code as a fixed predictor. Within each social disconnection intervention that involved friends, we computed simple correlations to examine the relationship between relationship quality and affective and cognitive state.

De-identified data and code to reproduce all analyses are available on the Open Science Framework: https://osf.io/vbg86/.

## Results

### Preliminary analyses

**Fig. 1:**
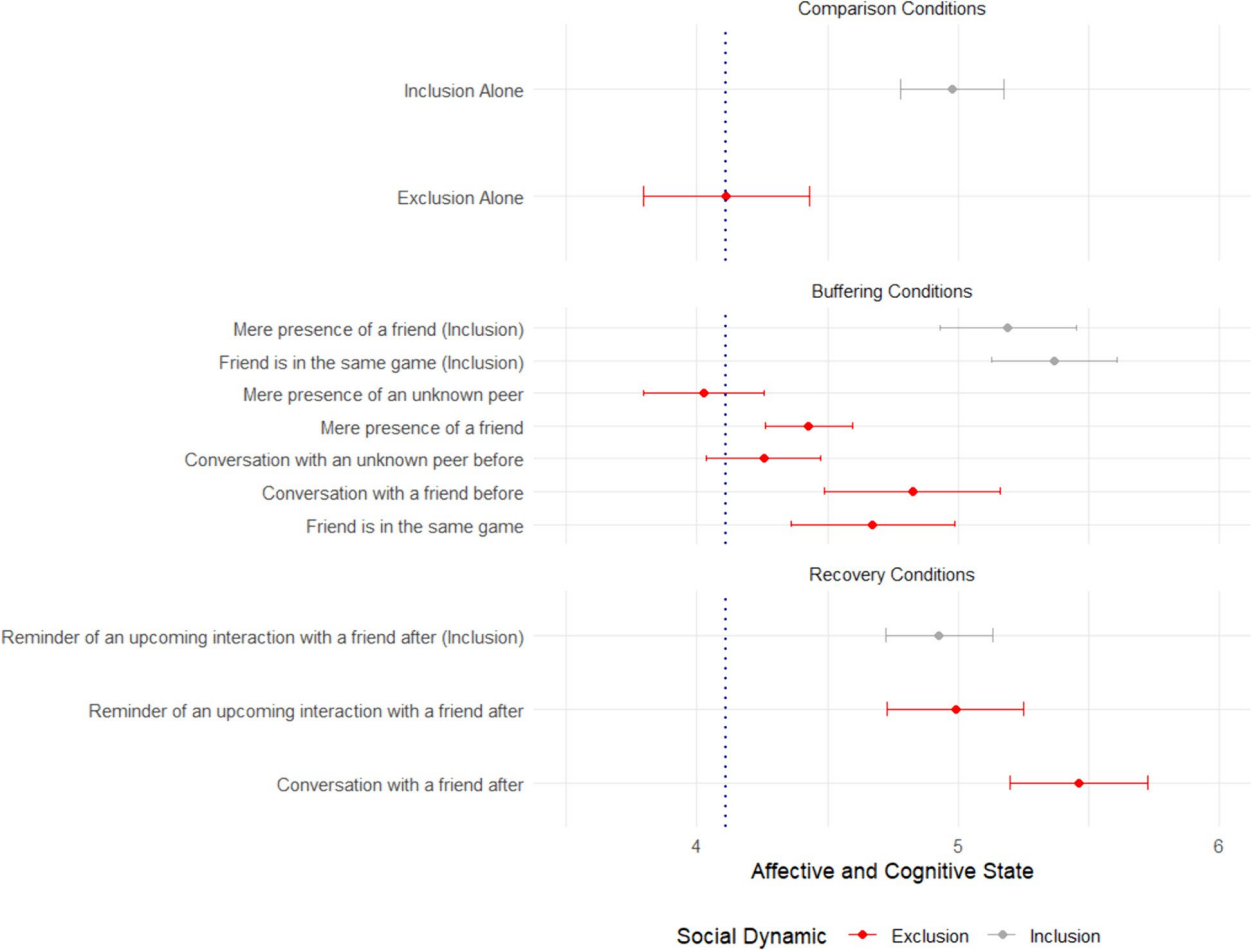
Affective and Cognitive State as a Function of Social Disconnection Intervention. Higher values indicate greater levels of affective and cognitive state, an aggregated measure of positive mood, a sense of belonging, perceived control, and social comfort. The blue dashed vertical line represents the actual observed mean response (not estimated marginal mean) to exclusion in the alone condition, which serves as a benchmark to assess the efficacy of the social disconnection interventions. Error bars represent 1 Standard Error above and below raw means. Not all social disconnection interventions have an inclusion condition

We aimed to replicate past work showing that one-person exclusion alone leads to negative affective and cognitive state [93, 94]. In our *alone* comparison conditions (top two bars in Fig. 1), we successfully replicate past work, and demonstrate the negative affective and cognitive consequences of experiencing *exclusion alone* as compared to *inclusion alone*. Experiencing social *exclusion alone* led to negative affective and cognitive consequences, as compared to *inclusion alone*, *t*(83) = − 4.461, *p <* .001, *d* = .97.

### Is there a buffering effect?

To examine the effect of our various social disconnection interventions in dampening the negative affective and cognitive consequences to exclusion, we first examined the effect of increasing the amount of social presence *prior* to experiencing social exclusion (i.e., *buffering*). Overall, three key findings emerge about the ingredients that give rise to effective buffering interventions.

First, the strongest buffering interventions consisted of participants having actual interactions with their friend before experiencing social exclusion. As shown in Fig. 1 (bars listed under “buffering”), having a *conversation with a friend before* experiencing social exclusion reduced the negative affective and cognitive consequences of social exclusion, as compared to experiencing *exclusion alone*, *t*(217.17) = 4.001, *p* < .001. Having a *conversation with a friend before* exclusion was not statistically significantly different from experiencing *inclusion alone*, *t*(184.52) = 1.652, *p* = .100. Moreover, the quality of the relationship predicted the affective and cognitive state for those who had a *conversation with a friend before* the experience of exclusion, *r*(33) = 0.409, *p* = .015.

Critically, the results show that having interactions with a friend, and not just simply being in the presence of a friend, might be an important component of the buffering intervention. Specifically, having a *conversation with a friend before* experiencing exclusion significantly reduced its negative affective and cognitive consequence as compared to simply having the *mere presence of a friend, t*(531.48) = 2.367, *p* = .018. Furthermore, having the *mere presence of a friend* was not significantly different than experiencing *exclusion alone*, *t*(387.21) = − 0.235, *p* = .814. The effect of *mere presence of a friend* was not predicted by relationship quality, *r*(132) = − 0.096, *p* = .272.

Second, our results highlight that conversations with friends may be a particularly effective component of buffering interventions. Having a *conversation with an unknown peer* did not offer conclusive evidence for buffering the affective and cognitive consequences of social exclusion as compared to experiencing *exclusion alone*. Our statistical model revealed a statistically significant effect*, t*(368.04) = 2.338, *p* = .020. However, when you look at the raw means (see Fig. 1), we see that the confidence intervals for having a *conversation with an unknown peer* and experiencing *exclusion alone* overlap, diminishing our confidence in the observed effect.

Third, and interestingly, having a *friend in the same game* while experiencing social exclusion did not significantly buffer against the negative affective and cognitive consequences of social exclusion as compared to experiencing *exclusion alone*, *t*(343.92) = 0.868, *p* = .386. The effect of *friend in the same game* was not predicted by relationship quality, *r*(51) = 0.208, *p* = .136. We discuss possible explanations for this boundary effect to buffering interventions in the Discussion.

Overall, the findings examining buffering interventions demonstrate that social interactions with friends and not just the mere presence are particularly important in ameliorating the negative consequences of social exclusion. They highlight the importance of dyadic conversations with friends in conferring buffering benefits, and that these benefits might be most pronounced when the conversations are with established social connections, especially high-quality relationships.

### Is there a recovery effect?

To examine how the social disconnection interventions may aid in the recovery from the negative consequences of social exclusion, we next examined the effect of social presence following the experience of social exclusion in Cyberball.

As shown in Fig. 1 (bars listed under “Recovery”), both recovery interventions significantly reduced the negative affective and cognitive consequence of experiencing social exclusion. Having a *conversation with a friend after* the experience of social exclusion reduced the negative affective and cognitive consequences of social exclusion, as compared to experiencing *exclusion alone*, *t(*244.00) = 7.359, *p* < .001. Having a *conversation with a friend after* exclusion was statistically indistinguishable from experiencing *inclusion alone*, *t*(202.18) = 1.548, *p* = .123. Moreover, relationship quality with the friend was not associated with the affective and cognitive state following a *conversation with a friend after* the experience of social exclusion, *r*(32) = 0.162, *p* = .361.

Moreover, our results demonstrate that even a symbolic representation (i.e., without direct physical engagement) of a future friend interaction facilitates the recovery following social exclusion. We find even a simple *reminder of an upcoming interaction with a friend after* exclusion significantly reduced the negative affective and cognitive consequence of experiencing social exclusion, as compared to experiencing *exclusion alone*, *t*(113.42) = 3.663, *p* < .001. Once again, the quality of the relationship was not associated with the affective and cognitive state following a *reminder of an upcoming interaction with a friend after* the experience of social exclusion, *r*(42) = 0.076, *p* = .626.

Why does a simple reminder of an upcoming interaction with a friend facilitate recovery? Could this effect be the result of simply reminding participants of their friend? It is noteworthy that in the presence of a friend condition, the friend was present throughout the entire experimental session, so participants were going to ostensibly reconnect with the friend at the end of the study. But the *mere presence of a friend* did not *buffer* against social exclusion, and both actual and symbolic interaction interventions significantly reduced negative affect compared to the presence of a friend condition. Participants who received a *reminder of an upcoming interaction with a friend after* inclusion reported higher affective and cognitive state than those who simply experienced *inclusion alone* in the game, *t*(226.25) = 3.300, *p* = .001.

Overall, these findings demonstrate that social interactions (real or symbolic) are particularly important in promoting recovery from negative consequences of social exclusion. This was the case whether there was an actual conversation, or the promise of an interaction, which demonstrates the power of symbolic reminders of social bonds in facilitating recovery from social exclusion. Importantly, the quality of the friendship does not seem to influence the magnitude of these effects. We discuss implications in the Discussion.

## Discussion

As soon as individuals detect that they are socially excluded, they immediately experience diminished mood, threatened fundamental needs, and social pain [106]. Arguably, such sensitivity to exclusion can be adaptive, functioning as a signal that something is wrong and motivating behaviors to repair potentially threatened ties [105–107]. Indeed, social exclusion is used to sanction norm-violating individuals [108]. At the same time, the experience of exclusion can be overwhelming, leading to maladaptive cognitions and emotions that may trigger behaviors that lead to antisocial behaviors, which ironically may perpetuate social alienation [109–112]. The present research is novel in its examination of how social disconnection interventions can mitigate the negative effects of social exclusion, which may enable individuals to more effectively adapt to their social environments.

### The efficacy of social disconnection interventions

In the present work, we varied whom participants interacted with (a friend vs. an unknown peer vs. alone), the nature of interpersonal engagement (having a face-to-face conversation vs. being reminded of an upcoming interaction vs. merely being in the presence of another), the timing of the social disconnection intervention relative to the experience of social exclusion (before vs. during vs. after). We find that not all social disconnection interventions are equally effective.

First, in our studies, whom the person interacted with emerged as a critical factor. As compared to being alone, having a conversation with a friend both before and after experiencing exclusion, regardless of the quality of the relationship, mitigated the affective and cognitive consequences of social exclusion. Notably, the benefit of interactions before experiencing social exclusion (i.e., buffering interventions) was related to relationship quality with said friend; the better the relationship, the better the outcome. However, the benefit of interactions after experiencing social exclusion was not related to the quality of the relationship. We speculate that when the conversation occurs *after* a threat*,* the interaction provides relief to a concrete and immediate social threat (i.e., the experience of social exclusion), and does so regardless of relationship quality. In contrast, when the conversation occurs *before* a threat, individuals may be uncertain about the nature and severity of potential threats (if any). Naturally, when relationship quality is lower, the confidence of the quality of future support remains in question. The perceived and received benefits of social support may be inconsistent or uncertain due to past experiences or ambiguity. This ambiguity can extend to the kind of support that might be needed in case of a threat, making it difficult to anticipate the level of support that will be available or necessary.

Were these benefits due to simply having a conversation with anyone? Our data suggest that the answer to this question is no. Specifically, our findings did not provide conclusive support that interacting with a previously unknown peer was effective in mitigating the affective and cognitive consequences of social exclusion. We note that the conversations between the friends and the unknown peers differed. Researchers have found that conversations between strangers are mostly spent finding common ground, whereas conversations between friends are mostly spent exploring new ground (see Speer, Mwilambwe-Tshilobo, Tsoi, Burns, Flak, & Tamir (2023) for a discussion) [113]. Thus, the conversations between the unknown peers were structured with the goal of facilitating connection, and used “getting to know you” type questions (e.g., “*What is your favorite class at Cornell?*”). In contrast, the conversations between friends were structured with the goal of prompting exploration, with the goal of deepening the bond, and incorporated questions from Aron’s “Fast Friends” procedure (e.g., “*What would constitute a perfect day for you?*”). While ecologically valid, this has implications for the interpretation of results. One possibility is that if the conversation methods were the same, a conversation with an unknown peer before might have buffered against the negative consequences of social exclusion. However, a more likely possibility is that, even if the conversation methods were the same, a conversation with an unknown peer would still not buffer. We suspect this is more likely due to uncertainty regarding the unknown peer’s capability and availability to provide future social support. The unknown peer is still a relative stranger who may never be seen again (for comparison, friends had a mean relationship length of nearly 20 months).

Second, the nature of interpersonal engagement of the social disconnection intervention also mattered. Having an interaction with a friend before (buffering) or after (recovery) mitigated the negative affective and cognitive consequences of social exclusion. Remarkably, even a simple reminder of an upcoming interaction with a friend after the experience of social exclusion was also powerful. Such effects may reflect the immediate consequences of activating the symbolic representation of the friend, such as positive affect, but may also reflect the anticipation of the future interaction. Critically, the mere presence of a friend or an unknown peer in the same room did not mitigate the negative consequences. This finding echoes previous research showing that merely being around others does not alleviate feelings of social disconnection [82].

Nonetheless, our present results extend previous research on how actual and imagined interactions regulate emotions and cognitions by demonstrating it in the context of social exclusion and underscore the importance of interactions (both real and imagined) in mitigating the negative affective and cognitive consequences of social disconnection. Future research should address the absence of recovery interventions that involved unknown peers. It remains to be seen whether conversations with unknown peers and reminders of an upcoming interaction with an unknown peer after the experience of social exclusion would be effective. Some research suggests that momentary vulnerabilities of social disconnection motivate individuals to form new bonds in the service of social connection [114]. Yet at the same time, there is mixed evidence on how short-term, momentary vulnerabilities of social disconnection either assist or hinder social processing. For example, some research suggests that after experiencing social exclusion, individuals are better at distinguishing between Duchenne (“real”) and non-Duchenne (“fake”) smiles, and that the experience increases the cone of direct gaze [115, 116]. Yet, other research has found that individuals are less likely to categorize faces as “happy” faces and less accurate in perceiving gaze direction after the experience of social exclusion [117, 118].

Of note, the social disconnection intervention where a friend was present in the actual social exclusion dynamic did not significantly mitigate the negative affective and cognitive consequences of social exclusion. In the friend in the same game condition, the friend’s behavior was impartial, equally directing their actions to the participant (50% of the time) and to the excluder (50% of the time). This intervention may not have been effective because the participant may have expected their friend to step up to do something about the social exclusion (e.g., rebuke the excluder by non-interaction). By continuing to engage with the excluder, individuals may have inferred that their friend tacitly accepted or tolerated their exclusion. The lack of an effect from this intervention is worthy of further investigation to understand inferences made by the participant for their friend’s behavior. Understanding the nuances of friend involvement *during* exclusionary events will be instrumental in shaping and informing the design of effective intervention strategies.

Third, timing mattered—a proposition consistent with other work on emotion regulation [119–121]. Although social disconnection interventions both before and following social exclusion aided in dampening the negative affective and cognitive consequences, recovery interventions were generally more robust than buffering interventions. More specifically, the mean affective and cognitive state of participants in the two recovery interventions were equal to and higher than inclusion alone. Earlier, we speculated that, for buffering interventions, the confidence of the quality of future support might not change when relationship quality is lower, whereas for recovery interventions, there is no question about receiving support. These findings are consistent with past work that has generally found more robust effects for recovery as compared to weak and inconsistent effects for buffering [81, 83–85]. Given our findings, it will be essential to investigate the nuanced role of timing in social disconnection interventions, such as examining the optimal points for inducing buffering and recovery effects. For example, future research could explore questions such as how close to the social exclusion event does the timing of interventions need to be.

The efficacy of interventions can be understood within an attachment framework, where a primary function of attachment figures is to restore affective and cognitive equilibrium after a threat has been encountered [53, 54, 62–67]. The realization of these benefits can be more challenging when individuals lack awareness or the ability to foresee future threats. These findings highlight the significance of implementing well-timed interventions in addressing the affective and cognitive toll of loneliness and social isolation.

### Implications for public health interventions, practices, and policies

Addressing public health concerns arising from social disconnection requires a consideration of the *structure* (how relationships are organized, the frequency of interactions), *function* (what roles relationships and interactions serve, such as social support), and *quality* (the level of satisfaction and fulfillment, which can vary across situations and relationships) [122]. In particular, a strong structural foundation, such as the proximity, contact, and presence of others, has downstream consequences on the function and quality of social relationships [20].

Building on previous work that suggests that social disconnection interventions should focus on strategies that increase opportunities for social contact and on cognitions that lead to social avoidance and withdrawal [29, 30], we manipulated factors that affect structure, function, and quality: (1) whom participants interacted with (alone vs. an unknown peer vs. a friend), influencing structure and quality, (2) the nature of interpersonal engagement (a face-to-face conversation vs. a reminder of an upcoming interaction vs. mere presence), influencing function and quality, and (3) the timing (before vs. during vs. after), influencing structure, function, and quality. Our research highlights the potential benefits of tailoring social disconnection interventions to leverage existing friendships and underscores the significance of fostering, encouraging, and nurturing relationships. Furthermore, our findings hold particular relevance for various organizations, including schools, colleges, and medical and occupational institutions. Implementing frequent, positive, and structured interactions in these settings may effectively promote social connection and reduce individuals’ vulnerability to social slights.

Our findings suggest social disconnection interventions that involve direct or symbolic interactions with friends is crucial for mitigating the detrimental effects of social exclusion. Specifically, having individuals engage in face-to-face conversations with friends before or after the threat of social exclusion, and activating symbolic representations of friends through reminders of upcoming interactions after, mitigate the negative affective and cognitive consequences of social exclusion. Integrating these interventions into public health interventions, practices, and policies can provide a proactive approach to primary prevention by targeting momentary vulnerabilities of social disconnection prior to the onset of loneliness and social isolation.

An advantage of the social disconnection interventions described here is that they have a low barrier to entry and are focused on momentary vulnerabilities of social disconnection. Part of the low barrier to entry is that the interventions do not require direct visible support that may be more difficult to provide. Direct visible support (e.g., “My advice to you is to...”) as compared to indirect invisible support (e.g., cooking dinner, hanging out) often has unintended consequences [123]. Bolger et al. have theorized that invisible or indirect social support, which recipients may not notice, or even interpret as support, leads to better outcomes. This is partly because direct social support is often associated with feelings of indebtedness or inequity [124].

Our current work primarily centers on young people, a group who is “at a high risk of feelings of loneliness but are least likely to take action” [11]. However, it will be equally important to examine how the effects of these social disconnection interventions vary across different groups of people who face loneliness and social isolation, including older populations with smaller but denser social networks, individuals from diverse racial and ethnic backgrounds, and the intersection of these factors [125–128]. These considerations will be essential for a comprehensive understanding of social disconnection interventions and their potential impact on public health.

## Conclusions

Our findings underscore the profound impact that seemingly small interactions can have in mitigating the negative affective and cognitive consequences of social exclusion. In fact, even the mere idea of such interactions can offer substantial benefits. Just as Samuel Taylor Coleridge eloquently expressed, “friendship is a sheltering tree” [12]. Friendships, much like the branches of a sturdy tree, offer protection, comfort, and security in the face of life’s challenges and uncertainties. They provide a highly effective and cost-efficient primary prevention intervention strategy to combat loneliness and social isolation.

### Supplementary Information

**Supplementary material 1.**

## Data Availability

De-identified data and code to reproduce all analyses are available on the Open Science Framework: https://osf.io/vbg86/.

## Abbreviations

IDA: Integrative Data Analysis
MLM: Multilevel Modeling

## Footnotes

1 Other names for IDA include “mega-analysis.”

2 The total percentage exceeds 100% because participants were able to select multiple identities.

3 The face-to-face conversation between friends and unknown peers differed by design. The conversation between the unknown peers were structured with the goal of facilitating social connection, and thus had “getting to know you” type questions (e.g., “*What is your favorite class at Cornell?*”). In contrast, the conversation between friends were structured with the goal of prompting exploration and had the goal of deepening the bond, and thus used Aron’s “Fast Friends” questions (e.g., “*What do you value most in a friendship?*”).

4 We do not treat relationship quality with friends as an additional social context variable because we do not experimentally manipulate this factor.

5 All participants in Study 2, Study 3, Study 4, and Study 5 answered questions regarding a friend. Participants in Study 1 did not answer these questions. Study 2 recruited participants alone and with a friend. Participants who were recruited alone (i.e., conversation with an unknown peer before (Study 2), mere presence of an unknown peer (Study 2)) answered these questions about their best friend as opposed to a friend they came into the study with.
