# Supplementary material for: Mitigating the affective and cognitive consequences of social exclusion: an integrative data analysis of seven social disconnection interventions
Source: BMC Public Health. 2024 May 7;24:1250. doi: 10.1186/s12889-024-18365-5 (PMC11075311; doi:10.1186/s12889-024-18365-5)
Supplement: Supplementary file 1 — Supplementary material 1. [file 12889_2024_18365_MOESM1_ESM.docx]

**Supplemental Materials**

**Overview of Study Materials**

Below, we provide details about the methods for each study in our integrative data analysis.

**Study 1**

Participants were randomly assigned to experience exclusion (i.e., one player never threw the ball to the participant while the other player threw the ball to the participant 50% of the time) or inclusion (i.e., both players threw the ball 50% of the time to the participant and 50% of the time to the other player) in Cyberball. All participants completed the experiment alone (i.e., there were no other participants in the room with them). These conditions served as benchmarks from which to assess the effect of our social disconnection interventions. This study utilized a Social Dynamic (Exclusion alone vs. Inclusion alone) between-subjects design.

**Affective and Cognitive State**. Affective and Cognitive State was assessed immediately after participants played Cyberball. Reliability for all items was high: Mood (“S*ad/Happy,” “Friendly/Unfriendly,” “Angry/Pleasant”*; alpha = .856). Belongingness (“*Disconnected/Connected*,” “*I belong/I don’t belong*,” “*Like an outsider/Like an insider*”; alpha = .792). Control (“*Powerless/Powerful*,” “*I have control/I lack control*,” “*Uninfluential/Influential*”; alpha = .790). Social Comfort (“*Uneasy /At ease*,” “*Comfortable/Uncomfortable*,” “*Awkward/Not awkward*”; alpha = .832). Overall, reliability was high. Cronbach’s alpha for all 12 items was .934.

**Study 2**

Participants were recruited to come in alone or with a friend. If the participant was recruited to come in alone, they were randomly assigned to either a partner (a confederate) with whom they would have a positive interaction (Conversation with an unknown peer before), or an unknown peer (a confederate) with whom they would not interact with at all (Mere presence of an unknown peer). All participants then experienced exclusion in Cyberball (i.e., one player never threw the ball to the participant while the other player threw the ball to the participant 50% of the time). This study implemented a three Social Presence condition (Conversation with an unknown peer before, Mere presence of an unknown peer, Mere presence of a friend) between-subjects design.

***Conversation with an unknown peer before.*** Participants completed the study with an unknown peer (a confederate) whom they did not know previously. Participants were told, “*Since this is the “Friend Study,” we assigned everyone a “Partner” based on his or her answers in the pretest. Looking at your answers, we decided that you two are very compatible. That’s why you are going to participate in this study together. To get to know each other a little bit more, what don’t you each ask and answer these questions*.” Participants were provided with a list of seven “getting to know you” questions (i.e. “*What is your name?”, “What year are you?”, “How old are you?”, “Where are you from?”, “What are you majoring in?”, “**What is your favorite class at Cornell?”, and “Tell one unique thing about yourself.”*). Following the conversation, participants were sat in a private cubicle and answered an initial mood measure and questions about the other person in the study room—the unknown peer. Participants then played Cyberball. During the game, the unknown peer was in another private cubicle in the same experimental room.

***Mere presence of an unknown peer.*** Participants completed the study with an unknown peer (a confederate) whom they did not know previously. If participants started talking to each other, the research assistant asked them to stop talking. Participants were sat in a private cubicle and answered an initial mood measure and questions about the other person in the study room—the unknown peer. Participants then played Cyberball. During the game, the unknown peer was in another private cubicle in the same experimental room.

***Mere presence of a friend.*** If participants were recruited with a friend, the researcher asked the pair how long the participants knew each other. Participants were sat in a private cubicle and answered an initial mood measure and questions about the other person in the study room—their friend. Participants then played Cyberball. During the game, their friend was in another private cubicle in the same experimental room.

**Initial mood.** Initial mood was assessed once participants were sat in a private cubicle prior to start Cyberball. Baseline Mood (“*Sad/Happy*,” “*Friendly/Unfriendly*,” “*Angry/Pleasant*”; alpha = .828).^[[1]](#footnote-1)^

**Interpersonal impressions.** Participants answered questions about their perceptions and feelings toward the person present in the study environment, which varied based on the experimental conditions (i.e., Conversation with an unknown peer before, Mere presence of an unknown peer, Mere presence of a friend), before playing Cyberball. The questionnaire included questions on willingness to collaborate on future tasks (*“How much would you enjoy working with this person on a future task?”*), closeness (“*How close do you feel to this person?*”), similarity (“*How similar do you feel to this person?*”), liking (“*How much do you like this person?*”), understanding *(“How well do you think this person can understand the kind of person you are?”*), acceptance (*“To what extent do you think this person will accept you?”),* trust *(“To what extent do you think you can trust this person?”*), positivity (*“How positive do you feel towards this person?”),* and negativity (“*How negative do you feel towards this person?”).* These items were answered on a 1 (*Not at all*) to 7 (*Very*) scale, with each item referring specifically to the question being answered (e.g., “*Not at all Close*,” “*Very Positive*”). Cronbach’s alpha for the entire scale was high (alpha = .954).

**Affective and Cognitive State.** Affective and Cognitive State was assessed was immediately after Cyberball. Mood (“*Sad/Happy*,” “*Friendly/Unfriendly*,” “*Angry/Pleasant*”; alpha = .863). Belongingness (“*Disconnected/Connected*,” “*I belong/I don’t belong*,” “*Like an outsider/Like an insider*”; alpha = .853). Control (“*Powerless/Powerful*,” “*I have control/I lack control*,” “*Uninfluential/Influential*”; alpha = .755). Social Comfort (“*Uneasy/At ease*,” “*Comfortable/Uncomfortable*,” “*Awkward/Not awkward*”; alpha = .769). Cronbach’s alpha for all 12 items was .923.

**Study 3**

Participants were recruited to come to the study with a friend. Once participants arrived, they were welcomed and sat in their own private cubicle. All participants were made aware of the possibility that one of the other players could be their friend (“*In this session, you will play a ball game with two other people – one of them could be your friend*”). All participants played Cyberball with their friend next to them in their own private cubicle. Once participants were seated in their private cubicle, they created a Study ID for themselves and answered a question about how they referred to their friend (“*Please type in the name you mostly strongly associate with your friend (e.g., first name, nickname)*”). Participants then answered a questionnaire unrelated to the present study. Following these tasks, participants played Cyberball. Participants were randomly assigned to either play Cyberball with their friend merely present or play with their friend in the game (i.e., the label of one of the players in the game referred to the name of their friend). Additionally, participants were randomly assigned to experience either exclusion (i.e., one player never threw the ball to the participant while the other player threw the ball to the participant 50% of the time) or inclusion (i.e., both players threw the ball 50% of the time to the participant and 50% of the time to the other player) in Cyberball. This study implemented a 2 Social Dynamic (Exclusion, Inclusion) x 2 Social Presence (Friend in the Game, Mere presence of a Friend) between-subjects design.

***Friend is in the same game.*** In the game, their friend threw the ball 50% of the time to the participant and 50% of the time to the other player. The name provided by the participants for their friend was used to designate one of the players in the game—this player was always inclusive (i.e., always threw the ball to the participant 50% of the time). During the game, their friend was in another private cubicle in the same experimental room.

***Mere presence of a friend.*** During the game, their friend was in another private cubicle in the same experimental room.

**Affective and Cognitive State.** Affective and Cognitive State Items was immediately assessed after the Cyberball game. Cronbach’s alpha for each individual subscale was high: Mood (“*Sad/Happy*,” “*Friendly/Unfriendly*,” “*Angry/Pleasant*”; alpha = .855). Belonging (“*Disconnected/Connected*,” “*I belong/I don’t belong*,” “*Like an outsider/Like an insider*”; alpha = .861). Control (“*Powerless/Powerful*,” “*I have control/I lack control*,” “*Uninfluential/Influential*”; alpha = .791); Social Comfort (“*Uneasy/At ease*,” “*Comfortable/Uncomfortable*,” “*Awkward/Not awkward*”; alpha = .824). Cronbach’s alpha for these 12 items was .936. ^[[2]](#footnote-2)^

**Study 4**

Participants were recruited to come to the study with a friend. At the start of the study, participants were told that they would play a game with other people but end the study session by have an interaction with their friend. Specifically, they were told “*In this session, you will play a game with two other people. Depending on the condition to which you have been assigned, one of the players may or may not be your friend.^[[3]](#footnote-3)^ After you play the game, you’ll answer some questions and perform a categorization task. Finally, you and your friend will participate in another activity*.” Participants were then seated at their own private cubicle. During the game, participants experienced either exclusion (i.e., one player never threw the ball to the participant while the other player threw the ball to the participant 50% of the time) or inclusion (i.e., both players threw the ball 50% of the time to the participant and 50% of the time to the other player) in Cyberball. This study utilized a Social Dynamic (Exclusion Alone vs. Inclusion Alone) between-subjects design.

**Reminder of an upcoming interaction with a friend after.** After playing Cyberball, participants were reminded that they would have an upcoming interaction with their friend. The activation of the friend representation was simple reminder, “*Before you participate in an activity with your friend, you will complete a few questionnaires and perform a cognitive task. Please answer the following questions based on how you feel RIGHT NOW*.” During the game, their friend was in another private cubicle in the same experimental room.

**Affective and Cognitive State.** After they were reminded of their upcoming interaction with their friend, Affective and Cognitive State was assessed. Cronbach’s alpha for each individual subscale was high: Mood (“*Sad/Happy*,” “*Friendly/Unfriendly*,” “*Angry/Pleasant*”; alpha = .767). Belongingness (“*Disconnected/Connected*,” “*I belong/I don’t belong*,” “*Like an outsider/Like an insider*”; Alpha = .724). Control (“*Powerless/Powerful*,” “*I have control/I lack control*,” “*Uninfluential/Influential*”; alpha = .695). Social Comfort (“*Uneasy/At ease*,” “*Comfortable/Uncomfortable*,” “*Awkward/Not awkward*”; alpha = .714). Overall, Cronbach’s alpha for all 12 items was .862.

**Study 5**

Participants were recruited to come to the study with a friend. Participants were randomly assigned to have a conversation with their friend before experiencing exclusion, have a conversation with their friend after experiencing exclusion, or merely have their friend be present. This study implemented a 3 Social Presence (Conversation with a Friend Before, Conversation with a Friend After, Mere Presence of Friend) between-subjects design.

**Conversation Task.** For participants who had a conversation with a friend, the sequence of the conversation task varied depending on whether participants had a conversation with a friend before or had a conversation with a friend after the experience of exclusion. Regardless of timing, the conversation task remained the same.

Participants were provided with a list of six questions (i.e., “*What would constitute a perfect day for you?*”, “*If you could wake up tomorrow having gained any one quality or ability, what would it be?*”, “*What was the best gift you ever received and why?*”, “*What is your favorite holiday? Why?*”, “*What do you value most in a friendship?*”, “*Share with your partner an embarrassing moment in your life*.”). The questions were drawn from Aron’s “Fast Friends” procedure, ^(1)^ which was originally designed to facilitate closeness by exploring conversational topics that are less likely to arise naturally in typical interactions. Each participant took turns asking their friend two questions from the list. This resulted in a total of four questions asked and answered, as participants also answered their own questions that they had asked. Participants switched roles after each question. This structured approach ensured that participants had the opportunity to engage in self-disclosure by both asking and answering questions during the conversation.

***Conversation with a friend before***. First, participants completed the conversation task and answered the Affective and Cognitive State measure. Next, participants played Cyberball, and completed Affective and Cognitive State measure. Then, participants selected and answered a neutral question (i.e., *“Describe your bedroom at home.”, “Write about your typical commute to school.”, “Write about your recent grocery shopping.”*) and completed the Affective and Cognitive State measure. During the game, their friend was in another private cubicle in the same experimental room.

***Conversation with a friend after***. First, participants answered a neutral question (i.e., *“Describe your bedroom at home.”, “Write about your typical commute to school.”,* or *“Write about your recent grocery shopping.”*) and completed the Affective and Cognitive State measure. Next, participants played Cyberball, and completed Affective and Cognitive State measure. Then, participants completed the conversation task and answered the Affective and Cognitive State measure. During the game, their friend was in another private cubicle in the same experimental room.

***Mere presence of a friend.***  First, participants selected and answered a neutral question (i.e., *“Describe your bedroom at home.”, “Write about your typical commute to school.”,* or *“Write about your recent grocery shopping.”*) and completed the Affective and Cognitive State measure. Next, participants played Cyberball, and completed Affective and Cognitive State measure. Then, participants selected and answered another neutral question from the same list as before (i.e., *“Describe your bedroom at home.”, “Write about your typical commute to school.”,* or *“Write about your recent grocery shopping.”*) and completed the Affective and Cognitive State measure. During the game, their friend was in another private cubicle in the same experimental room.

**Affective and Cognitive State.** Mood (“*Sad/Happy*,” “*Friendly/Unfriendly*,” “*Angry/Pleasant*”; alpha T1 = .715, T2 = .829, T3 = .878); Belongingness (“*Disconnected/connected*,” “*I belong/I don’t belong*,” “*Like an outsider/Like an insider*”; alpha = .573; T2 = .841, T3 = .858); Control (“*Powerless/Powerful*,” “*I have control/I lack control*,” “*Uninfluential/Influential*”; alpha = .655; T2 = .781; T3 = .810); Social Comfort (“*Uneasy /At ease*,” “*Comfortable/Uncomfortable*,” “*Awkward/Not awkward*”; alpha T1 = .553; T2 = .768; T3 = .778). Cronbach’s alpha for all 12 items was T1 = .823; T2 = 925; T3 = .935. ^[[4]](#footnote-4)^

**Summary of Personality Characteristics Across Studies**

| **Rejection Sensitivity Questionnaire** | | | | | |
| --- | --- | --- | --- | --- | --- |
| **Study 1 (Recruited Alone)** | **Study 2 (Recruited Alone)** | **Study 2 (Recruited with Friend)** | **Study 3 (Recruited with Friend)** | **Study 4 (Recruited with Friend)** | **Study 5 (Recruited with Friend)** |
| 10.00 (3.50) | 7.38 (2.96) | 6.48 (3.69) | 7.36 (3.37) | 7.33 (3.44) | 6.98 (3.38) |
| Note. The Rejection Sensitivity Questionnaire (RSQ) measures Rejection Sensitivity, the tendency to be concerned with and anxiously expect rejection. ^(3-4)^ We used a modified version of the RSQ that specifically asked participants about Rejection Sensitivity with friends. Scores on the RSQ can fall within a range of 1 to 36, with higher scores indicating heightened rejection sensitivity in social interactions. RSQ data are missing for 1 participant for Study 1, 3 participants for Study 2, 14 participants for Study 3, and 1 participant for Study 4, and 19 participants for Study 5. | | | | | |

| **Rosenberg Self-Esteem Scale** | | | | | |
| --- | --- | --- | --- | --- | --- |
| **Study 1 (Recruited Alone)** | **Study 2 (Recruited Alone)** | **Study 2 (Recruited with Friend)** | **Study 3 (Recruited with Friend)** | **Study 4 (Recruited with Friend)** | **Study 5 (Recruited with Friend)** |
| 36.24 (6.96) | 33.83 (5.88) | 33.29 (4.73) | 38.62 (6.36) | 39.82 (7.19) | 33.86 (5.39) |
| *Note*. The Rosenberg Self-Esteem Scale (RSES) measures a person’s level of self-esteem. ^(2)^ We used a modified version of the RSES such that participants responded using a 5-point Likert-type scale using anchors for “*Strongly Disagree*,” “*Disagree*,” “*Neither Agree nor Disagree*,” “*Agree*,” and “*Strongly Agree*,” with no numerical labels (the original scale uses a 4-point response scale). This modification aligns with recommendations to include a midpoint for the RSES, which allow participants to express neutral or mixed feelings about the self (see Donnellan & Rakhshani, 2023; see also Zayas, Wang, & McCalla, 2022). Scores on the modified RSES fall within a range of 1 to 50, with higher scores indicating higher self-esteem. | | | | | |

**Supplemental Analyses**

In our supplemental analyses, we control for Age, Gender, Race (White vs. Not), Rejection Sensitivity, and Self-Esteem.

In our study, participants were able to select more than one race and ethnicity, but for supplemental analyses, we dichotomized participants as White (if White was the only race and ethnicity that they selected) or not. We explicitly acknowledge the inherent limitations of categorizing participants as White vs. Not. It’s important to recognize the constraints of this categorization. The experience of being non-White is diverse and multifaceted, just as being White is not a uniform experience.

**Buffering Social Disconnection Interventions**

**Conversation with a Friend Before vs. Exclusion Alone**

We examined whether the effect of having a conversation with a friend before vs. exclusion alone remained after controlling for Age, Gender, Race, Rejection Sensitivity, and Self-Esteem. Indeed, it does, *t*(174.09) = 5.648, *p* <.001. See Supplemental Table 1.

| ***Supplemental Table 1*** |  |  |  |  |  |
| --- | --- | --- | --- | --- | --- |
| ***Predictor*** | ***Estimate*** | ***SE*** | ***df*** | ***t-value*** | ***p-value*** |
| (Intercept) | 2.899 | 0.5133 | 174.0899 | 5.648 | <.001 |
| Contrast (Conversation with a Friend Before vs. Alone) | 0.4581 | 0.1488 | 25.20373 | 3.079 | **.005** |
| Age | -0.0001919 | 0.0205 | 335.4815 | -0.009 | .993 |
| Gender (Male) | 0.04491 | 0.105 | 363.2869 | 0.428 | .669 |
| Race (White) | 0.01547 | 0.1005 | 364.7928 | 0.154 | .878 |
| Rejection Sensitivity | -0.01709 | 0.01508 | 338.384 | -1.133 | .258 |
| Self-Esteem | 0.05236 | 0.0083935 | 265.762 | 6.239 | <.001 |

Next, we examined whether the effect was moderated by Age, Gender, Race, Rejection Sensitivity or Self-Esteem. None of these factors significantly moderated the relationship. Specifically, the interaction effect with Age did not reach statistical significance, *F*(1, 407.16) = 0.0282, *p* = .867. The interaction effect with Gender was not statistically significant, *F*(1, 516.11) = 0.154, *p* = .695. The interaction effect with Race did not reach conventional levels of statistical significance, *F*(1, 404.97) = 3.377, *p* = .067. Rejection Sensitivity was not statistically significant, *F*(1, 499.15) = 0.0617, *p* = .804. Lastly, Self-Esteem was not statistically significant, *F*(1, 498.13) = 0.131, *p* = .717.

**Mere Presence of a Friend vs. Exclusion Alone**

We did not originally detect an effect of Mere Presence of a Friend vs. Exclusion Alone. This remains the case after controlling for Age, Gender, Race, Rejection Sensitivity, and Self-Esteem, it does, *t*(61.86) = -0.939, p = 3514. See Supplemental Table 2.

| ***Supplemental Table 2*** |  |  |  |  |  |
| --- | --- | --- | --- | --- | --- |
| ***Predictor*** | ***Estimate*** | ***SE*** | ***df*** | ***t-value*** | ***p-value*** |
| (Intercept) | 2.948 | 0.5223 | 275.324 | 5.645 | <.001 |
| Contrast (Mere Presence of a Friend vs. Alone) | -0.09071 | 0.09661 | 61.86085 | -0.939 | .351 |
| Age | 0.004019 | 0.02067 | 357.4107 | 0.194 | .846 |
| Gender (Male) | 0.059 | 0.1065 | 364.043 | 0.554 | .580 |
| Race (White) | 0.02226 | 0.1021 | 364.1293 | 0.218 | .828 |
| Rejection Sensitivity | -0.02546 | 0.0152 | 346.874 | -1.675 | .094 |
| Self-Esteem | 0.05036 | 0.008511 | 296.9623 | 5.917 | <.001 |

Given that we did not originally detect an effect, we do not further examine moderating factors.

**Conversation with an Unknown Peer Before vs. Exclusion Alone**

We were cautious about interpreting the effect of Conversation with an Unknown Peer vs. Exclusion Alone, given that that the confidence intervals overlap. Nevertheless, we present the model after controlling for Age, Gender, Race, Rejection Sensitivity, and Self-Esteem, *t*(53.53) = 2.390, *p* = .020. See Supplemental Table 3.

| ***Supplemental Table 3*** |  |  |  |  |  |
| --- | --- | --- | --- | --- | --- |
| ***Predictor*** | ***Estimate*** | ***SE*** | ***df*** | ***t-value*** | ***p-value*** |
| (Intercept) | 2.910 | 0.5174 | 185.8515 | 5.625 | <.001 |
| Contrast (Conversation with an Unknown Peer Before vs. Alone) | 0.4058 | 0.1698 | 53.52504 | 2.390 | .**020** |
| Age | 0.002679 | 0.02058 | 348.6484 | 0.130 | .897 |
| Gender (Male) | 0.04479 | 0.1055 | 363.352 | 0.424 | .672 |
| Race (White) | 0.01168 | 0.101 | 364.5204 | 0.116 | .908 |
| Rejection Sensitivity | -0.021 | 0.01507 | 330.3359 | -1.394 | .164 |
| Self-Esteem | 0.05209 | 0.008454 | 287.5769 | 6.161 | <.001 |

Given that we were cautious about the interpretation of the initial effect, we do not further examine moderating factors.

**Friend in the Game vs. Exclusion Alone**

We did not originally detect an effect of Friend in the Game vs. Exclusion Alone. This remains the case when controlling for Age, Gender, Race, Rejection Sensitivity, and Self-Esteem, *t*(24.23) = 1.790, *p* = .086. See Supplemental Table 4.

| ***Supplemental Table 4*** |  |  |  |  |  |
| --- | --- | --- | --- | --- | --- |
| ***Predictor*** | ***Estimate*** | ***SE*** | ***df*** | ***t-value*** | ***p-value*** |
| (Intercept) | 2.867 | 0.5054 | 156.8957 | 5.673 | <.001 |
| Contrast (Friend in the Game vs. Alone) | 0.202 | 0.1129 | 24.22727 | 1.790 | **.086** |
| Age | 0.002475 | 0.0205 | 299.2977 | 0.121 | .904 |
| Gender (Male) | 0.054 | 0.1061 | 364.7253 | 0.509 | .611 |
| Race (White) | 0.004203 | 0.1016 | 364.977 | 0.041 | .967 |
| Rejection Sensitivity | -0.02231 | 0.01484 | 234.2134 | -1.504 | .134 |
| Self-Esteem | 0.05295 | 0.008281 | 139.3403 | 6.394 | <.001 |

Given that we did not originally detect an effect, we do not further examine moderating factors.

**Recovery Social Disconnection Interventions**

**Conversation with a Friend After vs. Exclusion Alone**

We examined whether the effect of having a conversation with a friend after vs. exclusion alone remained after controlling for Age, Gender, Race, Rejection Sensitivity, and Self-Esteem. It does, *t*(14.78) = 6.042, *p* <.001. See Supplemental Table 5.

| ***Supplemental Table 5*** |  |  |  |  |  |
| --- | --- | --- | --- | --- | --- |
| ***Predictor*** | ***Estimate*** | ***SE*** | ***df*** | ***t-value*** | ***p-value*** |
| (Intercept) | 4.151 | 0.5358 | 163.8646 | 7.746 | <.001 |
| Contrast (Conversation with a Friend After vs. Alone) | 0.7222 | 0.1195 | 14.7846 | 6.042 | **<.001** |
| Age | -0.002114 | 0.01843 | 190.8945 | -0.115 | .909 |
| Gender (Male) | -0.1985 | 0.1194 | 197.817 | -1.662 | .098 |
| Race (White) | 0.2026 | 0.1149 | 194.3292 | 1.763 | .079 |
| Rejection Sensitivity | -0.01590 | 0.0174 | 191.3376 | -0.914 | .362 |
| Self-Esteem | 0.02520 | 0.008756 | 155.5282 | 2.878 | .005 |

Next, we examined whether the effect was moderated by Age, Gender, Race, Rejection Sensitivity, and Self-Esteem. The interaction effect with Gender (*F*(1, 233.00) = 0.227, *p* = .820) and the interaction effect with Race (*F*(1, 404.97) = 3.377, *p* = .620) did not emerge as statistically significant moderators. Age (*F*(1, 233.00) = 2.017, *p* = .045). The interaction effect with Rejection Sensitivity (*F*(1, 226.00) = -2.544, *p* = .012) and the interaction effect with Self-Esteem (*F*(1, 229.00) = 4.092, *p* < .001) did emerge as statistically significant moderators.

**Reminder of an Upcoming Interaction with a Friend After vs. Exclusion Alone**

We examined whether the effect of a reminder of an upcoming interaction with a friend vs. exclusion alone remained after controlling for Age, Gender, Race, Rejection Sensitivity, and Self-Esteem. It does, *t*(154.77) = 3.043, *p* = .003. See Supplemental Table 6.

| ***Supplemental Table 6*** |  |  |  |  |  |
| --- | --- | --- | --- | --- | --- |
| ***Predictor*** | ***Estimate*** | ***SE*** | ***df*** | ***t-value*** | ***p-value*** |
| (Intercept) | 4.015 | 0.602 | 74.43289 | 6.669 | <.001 |
| Contrast (Reminder of Interaction with a Friend After vs. Alone) | 0.3851 | 0.1266 | 154.7663 | 3.043 | **.003** |
| Age | -0.003233 | 0.01943 | 198.9122 | -0.166 | .868 |
| Gender (Male) | -0.193 | 0.1234 | 197.0494 | -1.563 | .120 |
| Race (White) | 0.2153 | 0.1198 | 197.8613 | 1.797 | .074 |
| Rejection Sensitivity | -0.01510 | 0.01833 | 198.7612 | -0.824 | .411 |
| Self-Esteem | 0.029 | 0.009324 | 198.9868 | 3.106 | .002 |

Next, we examined whether the effect was moderated by Age, Gender, Race, Rejection Sensitivity or Self-Esteem. None of these factors significantly moderated the relationship. Specifically, the interaction effect with Age was not statistically significant, *F*(1, 407.16) = 0.028, *p* = .867. The interaction effect with Gender was not statistically significant, *F*(1, 516.11) = 0.154, *p* = .695. The interaction effect with Race did not reach conventional levels of statistical significance, *F*(1, 404.97) = 3.377, *p* = .067. The interaction effect with Rejection Sensitivity did not reach statistical significance, *F*(1, 499.15) = 0.062, *p* = .804. Lastly, the interaction effect with Self-Esteem was not statistically significant, *F*(1, 498.13) = 0.131, *p* = .717.

**Supplemental References**

1. Aron A, Melinat E, Aron EN, Vallone RD, Bator RJ. The experimental generation of interpersonal closeness: a procedure and some preliminary findings. Pers Soc Psychol Bull. 1997;23(4):363–77.

2. Rosenberg M, Princeton University Press. Society and the adolescent self-image. Princeton: Princeton University Press; 1972.

3. Downey G, Feldman SI. Implications of rejection sensitivity for intimate relationships. Journal of Personality and Social Psychology. Journal of Personality and Social Psychology. 1996;70(6):1327-43.

4. Ayduk Ö, Mendoza-Denton R. A cognitive-affective processing system approach to personality dispositions.: rejection sensitivity as an illustrative case study. Handbook of Personality: Theory and Research. 2021;411-25

1. Belongingness, Control, and Social Comfort were not assessed at this point. [↑](#footnote-ref-1)
2. Study 3 included additional items that were not part of our Affective and Cognitive State aggregate, as not all studies included them: Self-esteem (“*Worthy/Unworthy*”, “*Inadequate/Adequate*”, “*Likeable/Unlikeable*”, alpha = .863); and Meaningful Existence (“*Significant/Insignificant*,” “*Purposeful/Purposeless*,” “*Frustrated/Satisfied*,” alpha = .796). Reliability for all 18 items (i.e., including the Self-Esteem and Meaningful Existence items) was .958. [↑](#footnote-ref-2)
3. In Study 4, the friend was never in the game. [↑](#footnote-ref-3)
4. Study 5 included additional items that were not part of our Affective and Cognitive State aggregate, as not all studies included them: Self-esteem (“*Worthy/Unworthy*”, “*Inadequate/Adequate*”, “*Likeable/Unlikeable*”, alpha T1= .649; T2= .844; T3= .768); Meaningful Existence (“*Significant/Insignificant*,” “*Purposeful/Purposeless*,” “*Frustrated/Satisfied*,” alpha T1= .631; T2 = .749; T3 = .756). Cronbach’s for all 18 items was T1 = .892, T2 = .951., and T3 =.950. [↑](#footnote-ref-4)
